# Two novel compound heterozygous *BMP1* mutations in a patient with osteogenesis imperfecta: a case report

**DOI:** 10.1186/s12881-017-0384-9

**Published:** 2017-03-04

**Authors:** Apiruk Sangsin, Chulaluck Kuptanon, Chalurmpon Srichomthong, Monnat Pongpanich, Kanya Suphapeetiporn, Vorasuk Shotelersuk

**Affiliations:** 1 Department of Orthopaedics, Faculty of Medicine, Chiang Mai University, Chiang Mai, 50200 Thailand; 2 Center of Excellence for Medical Genetics, Department of Pediatrics, Faculty of Medicine, Chulalongkorn University, Bangkok, 10330 Thailand; 3 Excellence Center for Medical Genetics, King Chulalongkorn Memorial Hospital, The Thai Red Cross Society, Bangkok, 10330 Thailand; 4 Department of Pediatrics, Queen Sirikit National Institute of Child Health, Bangkok, 10400 Thailand; 5 Department of Mathematics and Computer Science, Faculty of Science, Chulalongkorn University, Bangkok, 10330 Thailand; 6 Program in Bioinformatics and Computational Biology, Graduate School, Chulalongkorn University, Bangkok, 10330 Thailand; 7 Division of Medical Genetics and Metabolism, Department of Pediatrics, King Chulalongkorn Memorial Hospital, Sor Kor Building 11th floor, Bangkok, 10330 Thailand

**Keywords:** Osteogenesis imperfecta, *BMP1*, Next generation sequencing, Mutation analysis, Case report

## Abstract

**Background:**

Osteogenesis imperfecta (OI) is a collagen-related bone dysplasia leading to a susceptibility to fractures. OI can be caused by mutations in several genes including *BMP1*. It encodes two isoforms, bone morphogenetic protein 1 (BMP1) and mammalian tolloid (mTLD); both have proteolytic activity to remove the C-propeptide from procollagen.

**Case presentation:**

We report a Thai OI patient who had his first fracture at the age of three months. Using next generation sequencing, we successfully identified two novel compound heterozygous *BMP1* mutations. One mutation, c.796_797delTT (p.Phe266Argfs*25) affects both BMP1 and mTLD isoforms, while the other, c.2108-2A > G, affects only the BMP1 isoform. Preservation of the mTLD may explain the relatively less severe clinical phenotype in this patient. Intravenous bisphosphonate was given from the age of 8 months to 5 years. He was free from fractures for 9 months before discontinuation.

**Conclusion:**

This case expands the mutation spectrum of *BMP1*, strengthens the correlation between genotype and phenotype, and supports the benefits of bisphosphonate in OI patients with *BMP1* mutations.

## Background

Osteogenesis imperfecta (OI), a brittle bone disease, is a collagen-related bone dysplasia characterized by bone fragility leading to a susceptibility to fractures. Clinical manifestations of OI vary from intrauterine fractures and perinatal death to a mild form with few or no fractures [1]. Other features include short stature, bone deformities, joint laxity, dentinogenesis imperfecta, and blue sclerae. OI has been classified into autosomal dominant and recessive forms. More than 90% of OI patients are caused by mutations in *COL1A1* (Collagen Type I Alpha Chain) and *COL1A2* (Collagen Type II Alpha Chain)*,* inherited in an autosomal dominant manner. *COL1A1* and *COL1A2* encode alpha 1 and alpha 2 chains of type I collagen, respectively. Other inherited forms are rare and can be caused by mutations in different genes including *IFITM5* (Interferon Induced Transmembrane Protein 5) responsible for a dominant form, 14 genes for the recessive form, *BMP1* (Bone Morphogenic Protein 1)*, CRTAP* (Cartilage Associated Protein)*, CREB3L1* (CAMP Responsive Element Binding Protein 3 Like 1)*, FKBP10* (FKP506 Binding Protein 10)*, LEPRE1* (Leucine- And Proline-Enriched Proteoglycan 1)*, PLOD2* (Procollagen-Lysine, 2 –Oxoglutarate 5-Dioxygenase 2)*, PPIB* (Peptidylprolyl Isomerase B)*, SEC24D* (SEC24 Homolog A, COPII Coat Complex Component)*, SERPINF1* (Serpin Family F Member 1)*, SERPINH1* (Serpin Family H Member 1)*, SP7* (Sp7 Transcription Factor)*, SPARC* (Secreted Protein Acidic And Cysteine Rich)*, TMEM38B* (Transmembrane Protein 38B) and *WNT1* (Wnt Family Member 1) [2–4] and *MBTPS2* (Membrane Bound Transcription Factor Peptidase, Site 2) for the X-linked form [5].

Mutations in *COL1A1* and *COL1A2* result in primary structural or quantitative defects of type I collagen. The recessive forms of OI arise from defects in proteins that are responsible for post-translation modification of collagen, including defects in proteins responsible for folding and processing of collagen, and from defects in proteins that are necessary for ossification, mineralization or osteoblast development.

Proα1 and proα2 chains of type I collagen are post-translationally modified in the endoplasmic reticulum lumen into triple helix and then secreted into the extracellular matrix. After cleavage of their N and C-terminal propeptides, trimeric collagen molecules will be arranged into highly ordered collagen fibrils. *BMP1*, which is located on chromosome 8p21.3, encodes a protein with a role in proteolytic removal of the C-propeptide from procollagen. This crucial step is needed for the assembly of mature collagen monomers into fibrils [6]. Here we describe a Thai boy with OI who harbored two novel compound heterozygous *BMP1* mutations, c.796_797delTT (p.Phe266Argfs*25) and c.2108-2A > G.

## Case presentation

**Fig. 1:**
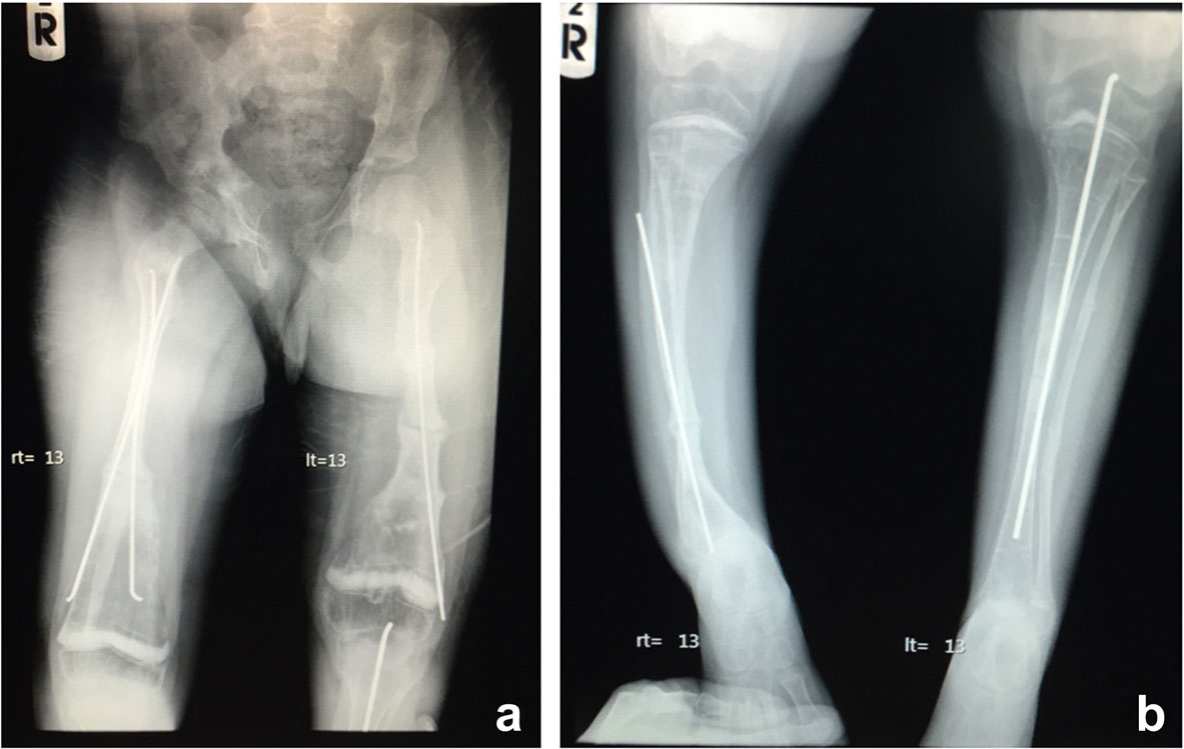
AP plain radiographs of lower extremities at the age of nine. **a** Both femoral AP radiographs reveal a nonunion at the shaft with shortening and varus deformity of the left femur. Intramedullary rods were used to stabilize the fractures. Generalized osteopenia is noted. **b** Both tibial AP radiographs reveal a nonunion, varus deformity, and shortening of the right tibia. Intramedullary rods were used to stabilize the fractures

Our patient was a 6-year-old boy, who was the second child of a non-consanguineous Thai couple. There was no family history of OI or other bone disorders. He was born at term with a birth weight of 3600 g (60^th^ centile). His early development was normal. He sustained the first fracture of his right arm at the age of three months from a minor trauma. Since then he had multiple fractures of various bones including both humeri, femora, tibiae, ulnae and radii, leading to a diagnosis of OI Sillence type III. Intravenous bisphosphonate was initiated at the age of 8 months; however, the fractures continued to occur once every few months and required many surgeries. At the age of 5 years, after he was free from fractures for 9 months, bisphosphonate was discontinued. His mental development was appropriate. He was able to walk until the age of six years, when a nonunion and muscle wasting of lower extremities occurred. Physical examination revealed deformities of both upper and lower extremities. However, no blue sclerae or dentinogenesis imperfecta was observed. Investigations revealed normal serum calcium, phosphorus and alkaline phosphatase levels. Plain radiographs of his long bones at the age of nine are shown in Fig. 1.

After informed consent was obtained, genomic DNA was extracted from leukocytes using a Puregene blood kit (Qiagen, Hilden, Germany). Sixteen known OI genes, *BMP1*, *COL1A1*, *COL1A2*, *CREB3L1*, *CRTAP*, *FKBP10*, *IFITM5*, *LEPRE1*, *PLOD2*, *PPIB*, *SERPINF1*, *SERPINH1*, *SP7*, *TMEM38B, WNT1,* and *MBTPS2,* were amplified from 200 ng of genomic DNA using the Truseq Amplicon Sequencing kit (Illumina, San Diego, CA). 286 amplicons which covered all the 226 exons (28 kb) of the target genes were sequenced by Miseq (Illumina, San Diego, CA) using 2x250 paired-end reads. All reads were aligned against the University of California Santa Cruz human genome assembly hg19 using Burrows-Wheeler Alignment software [7]. SNVs and Indel were detected by Miseq reporter software. Finally, possible disease causing variants were confirmed by PCR and Sanger sequencing. Segregation analysis was subsequently performed.

**Fig. 2:**
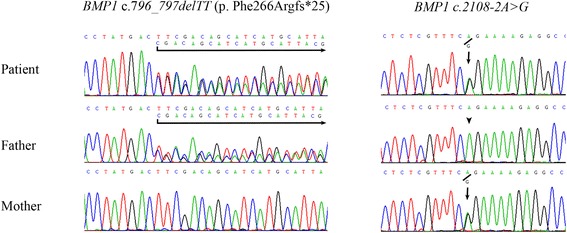
Mutation analysis. Direct sequencing shows that the proband is compound heterozygous for c.796_797delTT (p.Phe266Argfs*25) and c.2108-2A > G in the *BMP1* gene (NM_006129.4) while his father had c.796_797delTT (p. Phe266Argfs*25) and his mother had c.2108-2A > G

The targeted gene panel study using next generation sequencing of the proband revealed compound heterozygous mutations, c.796_797delTT (p.Phe266Argfs*25) and c.2108-2A > G in the *BMP1* gene (NM_006129.4). PCR followed by Sanger sequencing using genomic DNA of leukocytes from the patient and his parents confirmed that the patient was compound heterozygous for the mutations, while his father had c.796_797delTT (p. Phe266Argfs*25) and his mother had c.2108-2A > G (Fig. 2). Both mutations have never been previously described in Human Gene Mutation Database [8] or the Exome Aggregation Consortium database [9].

## Discussion

**Fig. 3:**
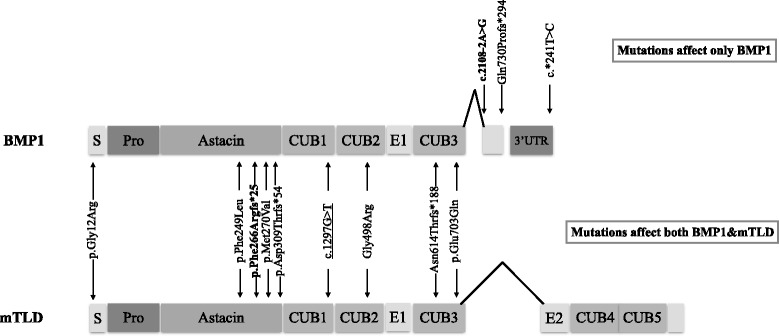
Map of the mutations in BMP1 and mTLD. Mutations shown between the two isoforms affect both BMP1 and mTLD, while those shown above BMP1 affect only BMP1. The mutations found in our patient are bolded. The underlined mutation is considered to cause exon 10 skipping

We identified a Thai patient with OI who had his first fracture at the age of three months. He was found to be compound heterozygous for c.796_797delTT (p. Phe266Argfs*25) and c.2108-2A > G mutations in the *BMP1* gene (Fig. 2). Both have never been reported. Adding this patient to the literature, there are currently 15 patients from 12 families (Table 1) who have an autosomal recessive form of OI resulted from mutations in the *BMP1*gene. Eight patients of six families were homozygous while the other seven patients of six families were compound heterozygous. Of these 12 families, 12 different mutations have been identified (Fig. 3).

**Table 1:** Reported OI patients with mutations in *BMP1*

Family	Number of cases	Zygosity	Mutation	Reference
1	2	Homozygous	c.747C > G (p.Phe249Leu)	[14]
2	2	Homozygous	c.34G > C (p.Gly12Arg)	[12]
3	1	Homozygous	c.34G > C (p.Gly12Arg)	[15]
4	1	Homozygous	c.*241 T > C	[13]
5	1	Homozygous	c.*241 T > C	
6	1	Homozygous	c.*241 T > C	
7	1	Heterozygous	c.*241 T > C; c.2107G > C (p.Glu703Gln)	
8	1	Heterozygous	c. 808A > G (p.Met270Val); c.1297G > T^*^	[16]
9	1	Heterozygous	c.925delG (p.Asp309Thrfs*54); c.1492G > A (p.Gly498Arg)	[11]
10	1	Heterozygous	c.34G > C (p.Gly12Arg); c.1839delC (p.Asn614Thrfs*188)	
11	2	Heterozygous	c.34G > C (p.Gly12Arg); c.2188dupC (p.Gln730Profs*294)	
12	1	Heterozygous	c.796_797delTT (p.Phe266Argfs*25); c.2108-2A > G	This report

*This variant caused exon 10 skipping

Processing of procollagen I C-terminal propeptide (PICP) is accomplished byfour BMP1-like proteinases: BMP1, mammalian tolloid (mTLD), and mammalian tolloid-like 1 and 2 (mTLL1 and 2). BMP1 and mTLD are resulted from the alternative splicing of the *BMP* gene, which is located on chromosome 8p21.3. The other two remaining members of this protein family, mTLL1 and mTLL2, are encoded by *Tll1* and *Tll2* located on chromosome 4q32.3 and 10q24.1, respectively. BMP1 has the highest PICP cleavage efficiency in vitro*,* followed by mTLD and mTLL1, while mTLL2 has no cleavage activity [10].

Of all the 12 *BMP1* mutations identified to date, nine affect both BMP1 and mTLD while the other three: c.2108-2A > G, Gln730Prof*294, and c*241 T > C affect only the BMP1 (Fig. 3). No mutations have been found in positions affecting only the mTLD. Those mutant alleles affecting only the BMP1 are expected to synthesize an intact mTLD, which has some PICP activity leading to a relatively milder form of OI. This hypothesis is true for the 11 families except Family 11 of Table 1, which harbored the c.34G > C (p.Gly12Arg) and c.2188dupC (p.Gln730Profs*294) mutations. Although the latter mutation affects only BMP1, two siblings of this family had severe bone fragility with more than a hundred fractures [11].

The conditions of the eight patients with both mutant alleles affecting both isoforms, no matter they are homozygous (Families 1, 2, and 3 of Table 1) or compound heterozygous (Families 8, 9, and 10 of Table 1), are relatively more severe. They had high frequency of fractures and severe bone deformities. For instance, the two patients with homozygous p.Phe249Leu mutation (Family 1 of Table 1), which is in the enzymatically active astacin domain affecting both BMP1 and mTLD, had very severe clinical presentation including severe bone fragility with fracture rate at 10-15/year, decreased bone mass, bone deformities, marked short stature and dysmorphic facial features.

Patients who were compound heterozygous with one mutant allele affecting only the BMP1 resulting in an intact mTLD (Families 7, 11, and 12 of Table 1) had relatively milder phenotypes. Our patient was compound heterozygous. One mutation, c.796_797delTT (p. Phe266Argfs*25) is an out-of-frame deletion in the astacin domain affecting both BMP1 and mTLD isoforms. The other mutation, c.2108-2A > G, is predicted to affect only the BMP1 isoform but preserve the mTLD (Fig. 3). This may explain the milder disease severity of our patient who had his first fracture at the age of three months, compared with patients whose mutations affect both BMP1 and mTLD.

Three patients (Families 4, 5, and 6 of Table 1) were homozygous for the c.*241 T > C. This mutation is predicted to affect only the BMP1 and preserve the activity of mTLD. They had the mildest phenotype. Their total number of fractures was 12, 16, and 18 times at the ages of 17, 22, and 28, respectively. In addition, two of them had the latest onset of the first fracture, which occurred at the ages of 2.5 and 4 years. Compared with a Canadian-French patient with compound heterozygous mutations, c.*241 T > C and p.Glu703Gln (Family 7 of Table 1) who had first fracture at birth and 12 total fractures at the age of eight, the three patients with homozygous c.*241 T > C had a later onset and lower rates of fractures. This finding could be explained by the fact that the p.Glu703Gln mutation in Family 7 affects the activity of both BMP1 and mTLD.

Interestingly, most of the OI patients with *BMP1* mutations have increased or normal bone mineral density (BMD), which is unusual for OI. Despite an increased BMD, some patients received bisphosphonate. After the treatment with bisphosphonate, two siblings with homozygous p.Gly12Arg had an increased BMD, an elevated urinary deoxypyridinoline excretion (a marker for osteoclastic activities) and a reduced fracture rate [12]. Intravenous bisphosphonate therapy in two Canadian-French patients (Families 6 and 7 of Table 1) with an elevated BMD also exhibited a similar result. One of them showed improvement in the shape of compressed vertebral bodies and a reduction in fracture rate [13]. Our patient was also given intravenous bisphosphonate and a decrease in fracture frequency was observed. Unfortunately, he did not undergo a BMD measurement. Whether bisphosphonate therapy in OI patients with *BMP1* mutations is useful awaits further studies.

## Conclusion

We described an OI patient with two novel compound heterozygous mutations in *BMP1*. One of the two is expected to preserve the mTLD isoform, which may lead to his relatively mild phenotype.

## Abbreviations

BMD: Bone mineral density
BMP1: Bone morphogenetic protein 1
mTLD: Mammalian tolloid
mTLL1: Mammalian tolloid-like 1
mTLL2: Mammalian tolloid-like 2
OI: Osteogenesis imperfect
